# Recommended Tests for the Self-Disproportionation of Enantiomers (SDE) to Ensure Accurate Reporting of the Stereochemical Outcome of Enantioselective Reactions

**DOI:** 10.3390/molecules26092757

**Published:** 2021-05-07

**Authors:** Jianlin Han, Alicja Wzorek, Karel D. Klika, Vadim A. Soloshonok

**Affiliations:** 1 Jiangsu Co-Innovation Center of Efficient Processing and Utilization of Forest Resources, International Innovation Center for Forest Chemicals and Materials, College of Chemical Engineering, Nanjing Forestry University, Nanjing 210037, China; hanjl@njfu.edu.cn; 2 Institute of Chemistry, Jan Kochanowski University in Kielce, Uniwersytecka 7, 25-406 Kielce, Poland; alicja.wzorek@ujk.edu.pl; 3 Molecular Structure Analysis, German Cancer Research Center (DKFZ), Im Neuenheimer Feld 280, D-69120 Heidelberg, Germany; 4 Department of Organic Chemistry I, Faculty of Chemistry, University of the Basque Country UPV/EHU, Paseo Manuel Lardizábal 3, 20018 San Sebastián, Spain; 5 IKERBASQUE, Basque Foundation for Science, Alameda Urquijo 36-5, Plaza Bizkaia, 48011 Bilbao, Spain

**Keywords:** self-disproportionation of enantiomers (SDE), enantiomeric analysis, molecular association, molecular chirality, enantiomeric excess

## Abstract

The purpose of this review is to highlight the necessity of conducting tests to gauge the magnitude of the self-disproportionation of enantiomers (SDE) phenomenon to ensure the veracity of reported enantiomeric excess (ee) values for scalemic samples obtained from enantioselective reactions, natural products isolation, etc. The SDE always occurs to some degree whenever any scalemic sample is subjected to physicochemical processes concomitant with the fractionation of the sample, thus leading to erroneous reporting of the true ee of the sample if due care is not taken to either preclude the effects of the SDE by measurement of the ee prior to the application of physicochemical processes, suppressing the SDE, or evaluating all obtained fractions of the sample. Or even avoiding fractionation altogether if possible. There is a clear necessity to conduct tests to assess the magnitude of the SDE for the processes applied to samples and the updated and improved recommendations described herein cover chromatography and processes involving gas-phase transformations such as evaporation or sublimation.

## 1. Introduction

As an integral part of our modern, innovation-driven industrialized society, the science of chemistry is undergoing dramatic changes, influencing the ways we learn, teach, conceptualize, and perform. One of the most noticeable signs of progress in the sciences is the exponential growth in the number of publications and their length, leading to ever-increasing pagination of established journals and the appearance of numerous new ones. It can be posited that this uncontrolled growth is inevitably accompanied by a decrease in the quality of experimental results. A major groundbreaking solution to the problem of questionable results was the implementation of supporting material documenting the analytical data and compound characterizations conducted in the study, a means not previously feasible for any given reader prior to the invention of the Internet. Nevertheless, the quality and integrity of the experimental data are still major concerns [1,2,3,4]. Over the last decade, systematic research into the phenomenon of the self-disproportionation of enantiomers (SDE) [5,6,7,8,9,10] has clearly revealed that the accuracy in reporting the stereochemical outcome of enantioselective reactions represents a major issue of concern in terms of data integrity and the understanding of the stereochemical mechanisms at play and the interpretation of the results. It has been unequivocally shown that any physicochemical treatment, including achiral chromatography [10,11,12,13,14,15,16,17,18,19,20,21,22,23,24,25,26,27,28,29,30,31,32,33,34,35,36,37,38,39,40,41,42,43] or even rotary evaporation [40,44], of a scalemic sample inevitably leads to fractionation of the material into portions of variable enantiomeric excess (ee), with consequent erroneous reporting of the stereochemical outcome if due care is not taken. Yet, there is a deficiency in the general understanding and appreciation of the SDE phenomenon, even though it has been known for a considerable length of time now. While the SDE is unavoidable, its effect on the accuracy of the data reporting can be mitigated by simple SDE tests, which may reveal the SDE magnitude as a function of the physicochemical conditions, as well as the range of possible error for the data under the given conditions. We believe that overall knowledge of the nature and manifestation of the SDE phenomenon has come of age and the time is right for the introduction of mandatory SDE tests to be part of the supporting documentation and a cultured practice of research chemistry, as it is clearly necessary [45,46,47]. In this article, we provide a short overview of the SDE phenomenon, cite key examples of extraordinary cases, and suggest simple generic SDE tests via achiral chromatography and sublimation, to be performed as an integral part of the experimental work submitted for publication when the work concerns scalemic samples that arise from enantioselective reactions or as a result of natural products isolation. The updated and improved recommendations for tests described in this work cover chromatography and processes involving gas-phase transformations such as evaporation or sublimation. While warnings and recommendations have also been issued in the past, clear protocols for conducting SDE tests have not been prominently featured or detailed.

## 2. Preview of the SDE

### 2.1. Origin and Purpose

As some readers may not yet be familiar with the SDE phenomenon, we briefly summarize here some of the most important principles of the SDE. The term SDE was rationally designed [29], and its linguistic etymology, as well as the pros and cons of its application, have been discussed [48]. While the SDE is a relatively new term, it actually describes rather “old”, quite well-known phenomena. For example, since 1895, or perhaps even earlier [49,50], chemistry practitioners knew that the crystallization of partially resolved samples of chiral compounds (also variously described as optically active, nonracemic, enantiomerically enriched, or scalemic) usually resulted in a precipitate of greater, and concomitantly, a mother liquor of lower, ee in comparison with the starting material, i.e., there was a re-distribution (disproportionation) of the enantiomers among the fractions. By analogy with chemical purification by crystallization, this process is most commonly called “optical purification”, or by a variety of other names involving optical/enantiomeric/chiral in combination with purification/enrichment/amplification [51,52,53]. Arguably, any combination of these aforementioned words does not describe correctly the outcome of a process producing simultaneously enantioenriched and -depleted fractions with no loss or gain in overall chirality. Accordingly, the introduction of appropriate terminology facilitating a chemically precise description of this type of physicochemical process was well overdue by the time that the SDE term was coined, though not only for reasons of scientific precision and methodological clarity. Due to the monotonously increasing accuracy of analytical methods, it became ever more apparent that processes resulting in the simultaneous formation of enantioenriched and -depleted fractions were not just limited to crystallization and, in fact, are rather ubiquitous with the SDE manifesting itself via virtually any type of physicochemical transformation.

### 2.2. Areas of SDE Manifestation

The areas of SDE manifestation can be divided into three major classes: physicochemical phase transitions, force field applications, and chromatography. We now briefly describe each of these in turn.

#### 2.2.1. Physicochemical Phase Transitions

Sublimation, a solid-to-gas phase transition, is, like crystallization, which will not be discussed since it is so well known, a classic method for the purification of crystalline organic compounds. However, SDE via sublimation (SDEvS)/evaporation (SDEvE) represents a major concern in regard to the accuracy of the experimental data, as the drying of reaction products under vacuum is usually an integral part of routine, reaction work-up procedures. Numerous examples of SDEvS have been reported [54,55,56,57,58] in the literature, strongly pointing to the necessity to control the ee integrity of samples by using an appropriate SDEvS test. Intriguingly, SDEvE, a liquid-to-gas phase transition, has even been observed by distillation [59,60,61,62], though convincing examples are extremely rare [63,64]. Among other potential SDE manifestations via phase transitions, liquid-to-liquid phase transition, as in extraction, can also be considered a possibility, and it is a process which plays a pivotal role in asymmetric catalysis using aqueous–organic bilayer conditions [65].

Sublimation, however, greatly differs in practice from recrystallization, as it is generally inconvenient to conduct it under equilibrium conditions for preparative purposes, as the gas phase is limited in how much mass it can contain, thus the occurrence of the SDE during sublimation primarily results from the different rates of transition from the solid state to the gaseous state of the racemic and enantiopure crystals, which is correlated to their thermodynamic stability. Particularly important for the kinetics of the sublimation process in practice are the manner in which the sample was prepared—mixed enantiopure crystals or mixed enantiopure and racemic crystals, crystal size and shape or amorphous, and origin of the samples if mixed. Confusion in the literature has resulted when attention has not been paid to this aspect [65]. It can be expected that the majority of compounds have euamotic points [57], i.e., they behave as racemic compounds by sublimation, just as the majority of compounds behave as racemic compounds by way of recrystallization. (Though it is interesting to note that sublimed material can adopt the alternate behavior [42,53,66,67].) Moreover, as long as there are no strong kinetic effects and the system is under quasithermodynamically controlled conditions, the correct state of crystals are used (i.e., the sample is deposited from a solvent or the same solvent is used if a mixture of racemic and enantiopure samples is prepared), the same crystal dimensions are present by grinding the sample to a powder, there are no seeding effects, adiabatic passage applies, and there is no changeover from racemic compound behavior to conglomerate behavior, then the following empiricisms, which are a re-wording of Jacques et al. [51], apply:

1. There is very likely to be a euamotic point, since the vast majority of compounds are likely to be racemic compounds by sublimation in concert with their crystallization behavior,
2. Below the euamotic point, the more volatile portion enriches the sublimate and conversely above it, and
3. The portion with the lower melting point (mp) is generally the more volatile (i.e., there is a correlation between mp and volatility) and which is typically the enantiomer.

Compounds that behave as conglomerates by sublimation, on the other hand, yield sublimates that are racemic.

#### 2.2.2. Force Field Applications

Unlike recrystallization and sublimation, SDE via force field (SDEvFF) deals with the static case where molecules remain in their original starting crystal structures and a force field enacts separation due to differing crystal densities. According to Wallach’s rule [49,50], for racemic compounds—representing more than 90% of crystalline organic compounds—the racemic crystals are packed comparatively more tightly than the enantiopure crystals, thus resulting in higher densities for the former. A quite remarkable example of separating a mixture of racemic crystals and enantiopure crystals due to their different densities was realized [68] for a mechanical mixture of racemic and enantiopure crystals of (*S*)-alanine by density gradient ultracentrifugation. Another demonstration of SDEvFF was reported [69] for a simple dispersion of chiral compounds suspended in specially prepared liquids, maximizing the difference in the densities of the racemic and enantiopure crystals. For example, in a mixture of chlorobenzene and bromobenzene as the suspension medium, the denser racemic crystals gravitated to the lower reaches of the system, while the lighter enantiopure crystals moved towards the upper reaches, allowing almost complete separation of the racemic portion from the enantiopure portion in the case of phenylalanine. Another very impressive example reported [70] using magnetic levitation to affect the SDE of ibuprofen, notable not just for the technique, but also for the fact that the enantiopure and racemic crystals of ibuprofen differ in density by only ~2%. Clearly, the cost effectiveness and outright operational convenience of these separations should be highly motivating for the development of practical large-scale enantiopurification procedures, especially in an industrial setting. Considering the overwhelming generality of the differences in crystal densities between racemic and enantiopure crystals, it is rather surprising that only a very limited number of reports on SDEvFF exist. One of the reasons might be because these reports seem to be almost completely unknown to the wider scientific community. We hope that the readers of this article will contribute to the due recognition and appreciation of this area of highly innovative research and its potential for unconventional means of enantiopurification [5,71]. In fact, the SDE has even found applications in conventional diastereomeric salt crystallization for the purposes of enantiopurification [72].

#### 2.2.3. Chromatography

SDE via chromatography (SDEvC) is arguably the major and the largest area of the SDE manifestation. The previous areas are all limited by crystallinity, volatility, and/or crystal density variance, properties possessed by less than ~10% of chiral organic compounds. In sharp contrast, on the other hand, practically all compounds can be subjected to chromatography, as the conditions that can be applied are virtually limitless. Therefore, whether compounds are crystalline or liquid, of low or high molecular weight, polar or non-polar, etc., they are likely to be amenable to chromatography. Notably, SDEvC has been observed for all manner of chromatographic techniques, such as high-pressure liquid chromatography (HPLC) [11,12,13,14,15,16,17], medium-pressure liquid chromatography (MPLC) [18,19,20,21], routine, gravity-driven column chromatography [22,23,24,25,26,27,28,29,30,31,32,33,34,35,36,37], flash chromatography [35,38,39,40], preparative thin-layer chromatography (PTLC) [10,41], and size-exclusion chromatography [42,43], as well as under a variety of conditions such as normal- and reverse-phase conditions and for various stationary phases [37,38]. Some noteworthy theoretical studies [15,73,74,75,76,77] have been conducted on SDEvC to describe the process. Interestingly, there is a clear correlation between the rising sophistication and availability of chromatographic instrumentation and the increasing reporting of SDEvC cases in the literature [78]. The sheer number of examples reported thus far, as well as the structural variety of compounds for which the SDE is highly expressed, overwhelmingly support the necessity for SDEvC tests to be a mandatory part of modern experimental procedures.

### 2.3. SDE-Phoric Groups

It is worth emphasizing again that the SDE is a ubiquitous phenomenon that is always occurring anytime an enantioenriched sample is subjected to any type of physicochemical treatment, or simply being exposed to conditions conducive to physicochemical transformations, such as sublimation during storage [10,66,79,80]. There are several parameters to describe or quantify the SDE, e.g., SDE range, SDE yield, maximum theoretical SDE yield, etc. [5,48], but by far the most important is the SDE magnitude (Δee), defined as [30]:

∆ee = ee_fraction with the highest ee_ − ee_fraction with the lowest ee_. (1)

**Figure 1:**
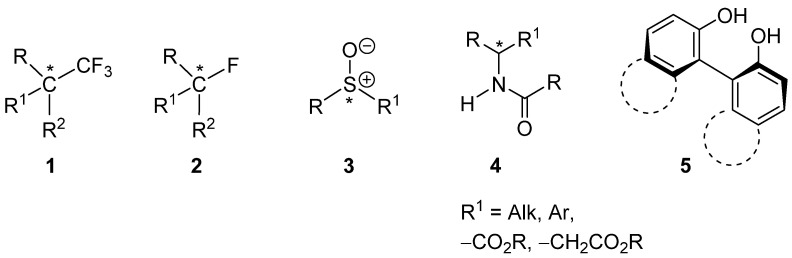
Examples of compounds **1**–**5** that contain SDE-phoric groups. The chiral center is marked with an asterisk in **1**–**4**, while axial chirality is present in **5**.

It should be noted that ∆ee can closely approach, but can never be equal to, 100%. Conversely, while the SDE magnitude can be below the level of detection, it is never equal to zero. Additionally, it is important to note that the ∆ee is not only a function of a particular compound’s structure but, to a great degree, also the conditions of the SDE experiment, such as the temperature, solvent/eluent used, impurities present, etc. Nevertheless, there is a significant degree of predictability of high SDE magnitude based purely on chemical structural features. From numerous literature data, it was deduced that molecules containing particular functional groups often have a propensity for exhibiting exceptionally high SDE magnitude, and such functional groups have been denoted as SDE-phoric groups [81]. Typically, the functional groups are strongly polarized and thus capable of forming strong intermolecular hydrogen [22,26,31,34,40,81] or halogen bonds [82,83], or undergoing intermolecular dipole–dipole interactions [11,38,84,85]. For example, the –CF_3_-containing groups in **1** (Figure 1) and the C–F-containing groups in **2**, unsurprisingly, due to their high electron withdrawing capability, can induce dipole–dipole interactions, as well as strongly enhancing the hydrogen bonding capability of other functional groups, such as OH [29,39,86] and NH [29,33]. Indeed, substances containing fluorine [86,87] are known to often express exceptionally high SDE magnitudes by chromatography [34], sublimation [88,89], and even distillation [59,60,61,62,63,64]. This aspect is particularly important considering the rapid growth in the number of chiral, fluorine-containing pharmaceuticals [90,91,92,93,94,95,96] and agrochemicals [97,98,99,100]. Sulfoxides **3**, due to the highly polarized S–O bond, are another example of an SDE-phoric group [26,71,85,101,102]. Of note, prazoles, S–O containing proton pump inhibitors that reduce the amount of acid produced in the stomach, are in the top 50 most prescribed drugs currently on the pharmaceutical market. Interestingly, both pantoprazole and lansoprazole [103] each contain both S–O and C–F groups.

Of particular note are the *N*-acyl amine-containing groups in **4** with an adjacent chiral center [19,20,22,30,81,104], a ubiquitous structural feature of numerous natural compounds and pharmaceutical drugs. When R^1^ = –CO_2_R or –CH_2_CO_2_R, the structures include α- [36,105,106] and β-amino [31,32] acid derivatives, respectively, such as peptides and peptidomimetics. There is no need to emphasize the exceptional hydrogen bonding properties of the N-acyl amine group in **4** and the obvious importance of this class of compounds in modern organic synthesis, medicinal chemistry, and drug design [107,108,109,110,111]. Finally, the 1,1′-bi-2-phenol structural unit present in **5** exhibits extraordinary hydrogen bonding properties and is responsible for the very high SDE magnitude often observed for these compounds [12,13,15,76,77,112,113,114,115,116]. For example, BINOL, possessing this structural feature, is an exceptional asymmetric catalyst [117] and known for large nonlinear effects [118,119,120,121] as a consequence of its outstanding SDE properties.

## 3. Pedagogic Examples from the Literature

The SDE literature has been well reviewed previously, including generally [5,47], for particular processes, e.g., sublimation [54] and chromatography [45,46,78,122], as well as for specific SDE-phoric groups, e.g., sulfoxides [71,102], fluorine-containing compounds [86,87,123,124,125], amides [102,126], and amino acids and their derivatives [105]. Of note, SDEvS has been much less reported, for the obvious reason that it is simply a less utilized technique compared to chromatography for routine purification. In this section, we draw attention to some notable examples of serendipitous SDEvS/E and SDEvC discoveries made during the course of regular research work to highlight various aspects of the SDE. We also speculate on how the SDE could impact reported results if researchers are ignorant of the SDE or simply lack scientific curiosity if they happen to encounter the SDE.

### 3.1. SDEvS

**Scheme 1:**
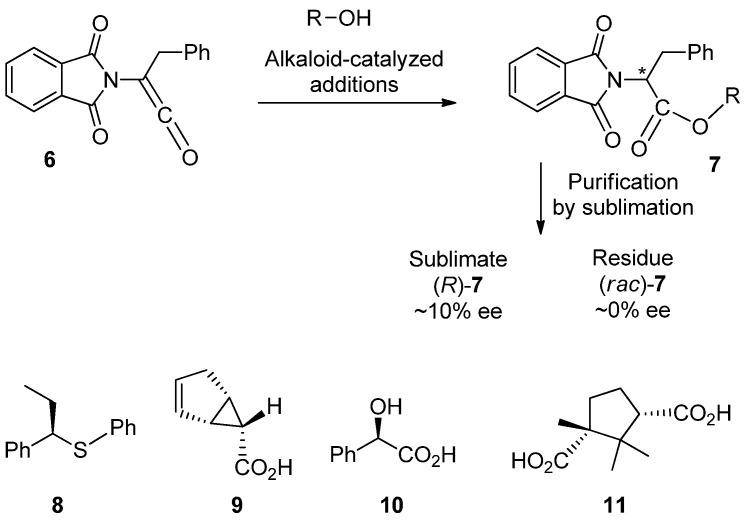
Examples of compounds **6**–**11** that exhibit SDEvS. The chiral center is marked with an asterisk in **7**.

As already pointed out, sublimation is a classical purification technique for organic compounds that has been routinely applied since the dawn of organic chemistry. Accordingly, it can be safely assumed that the purification of scalemates by sublimation has been performed countless times, especially, for example, during the isolation and purification of natural products. Surprisingly, therefore, it was not until 1959 when Dr. Gisela Pracejus [127] became the first pedant and thorough investigator curious enough to check the enantiopurity of both sublimed and residual portions after reaction products were subjected to routine purification by sublimation during a study of alkaloid-catalyzed addition reactions of alcohols to ketene **6** (Scheme 1). It was found that while the residue was chemically pure, it was a completely racemic product (*rac*)-**7**. In sharp contrast, the sublimed material **7** was enantioenriched to ~10% ee, indicating a substantial difference in the sublimation rates between (*rac*)-**7** and (*R*)-**7**. Obviously, if careful analysis of both sublime and residual material had not been conducted, the result of this work would be erroneously reported as disappointing, exhibiting no enantioselectivity and completely lacking as a promising opening approach for the catalytic asymmetric synthesis of amino acids.

Surprisingly, this extraordinary discovery did not register in the research conscience of practitioners and SDEvS was only “discovered” again in 1967 [128] for compound **8**, and then again a decade later in 1977 [129], for a series of naturally occurring compounds **9**–**11**. Even the publication of these results in the American Chemical Society’s *Journal of Organic Chemistry* in English was neither convincing nor helpful, as no one paid due attention to the accuracy of their experimental results, as further, new examples were not forthcoming until 1999 [44].

**Scheme 2:**
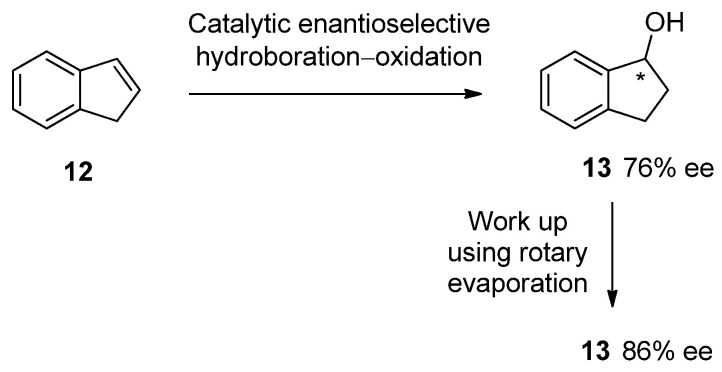
An example of a compound that exhibited SDEvE under routine rotary evaporation in the work-up following reaction. The chiral center is marked with an asterisk in the product **13**.

The most concerning case of unintentional SDEvE was encountered by Prof. John M. Brown [44] during work on the enantioselective hydroboration–oxidation of alkenes (Scheme 2). For the transformation of indene (**12**) to (*R*)-2,3-dihydro-1*H*-inden-1-ol (**13**), the authors found disturbingly low reproducibility with regard to the enantiopurity of product **13**. Meticulous examination of the experimental details revealed that the chemical yield and stereochemical outcome depended on length of exposure of product **13** to vacuum, in particular, removal by rotary evaporation of the reaction solvent. Aromatic alcohol **13** is particularly volatile, and the racemic portion of **13** can readily sublime, reducing the observed chemical yield and increasing the enantiopurity of the remainder. The fact that a routine work-up procedure such as evaporation of the reaction solvent resulted in significant alteration of the observed stereochemical outcome should be no less than alarming, and even disturbing, as it points to a very high probability that this kind of problem may have occurred frequently in the past. Nevertheless, this incident was not fully investigated and the paper has practically not been cited by other practitioners reporting on the enantioselective synthesis of volatile aromatic alcohols.

**Scheme 3:**
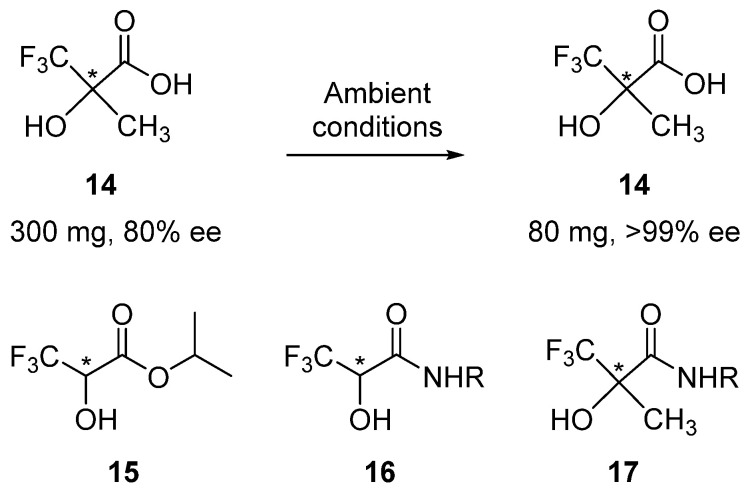
Examples of fluorinated analogs of lactic acid **14**–**17** that exhibit SDEvS. The chiral center is marked with an asterisk in **14**–**17**.

Another noteworthy example is the fluorinated analog of lactic acid **14** (Scheme 3). Due to the exceptionally high volatility of compound **14**, sublimation readily takes place at ambient temperature and pressure. In a typical experiment, 300 mg of acid **14** of 80% ee was spread over a Petri dish and left on a bench. After some 56 h, 80 mg of enantiopure **14** was left behind in the Petri dish! Measurement of the sublimation rates revealed [130,131] that racemic crystals of **14** sublimed about twice as fast in comparison to enantiopure crystals.

Similar sublimation behaviors were reported for other types of fluorinated lactic acid derivatives, e.g., ester **15** [42,55] and amides **16** [88] and **17** [89]. In general, the introduction of fluorine into an organic compound, in particular, a trifluoromethyl or perfluoroalkyl group, noticeably increases the volatility of the resulting derivative. Considering the ever-increasing research into the design and synthesis of biologically active fluorinated compounds [90,91,92,93,94,95,96,97,98,99,100,132,133], this property of fluorine-containing molecules should be taken very seriously. Thus, since many small organic compounds, especially fluorine-containing ones, possess sufficiently high volatilities, they are prone to the SDE during processes involving gas-phase transformations. Even during the course of solvent removal under vacuum [40,44] or sample drying, as well as the way in which the compounds are stored [10,66,79,80], the ee of scalemic samples can be altered. Obviously then, SDEvS/E represents a major concern with regard to the quality and reliability of reported stereochemical outcomes of catalytic enantioselective reactions, as well as for the isolation of natural products and the handling of pharmaceutically active intermediates [123,124,125]. Taking into account that rotary evaporation and drying under vacuum are an innate part of routine reaction work-up and analytical sample preparation procedures, it is impossible to proffer arguments against the necessity to conduct a simple SDEvS test to ensure the accuracy of one’s results. Hence, SDE tests for evaporation/sublimation should be considered obligatory.

### 3.2. SDEvC

As already mentioned, the SDEvC is the largest area of SDE manifestation. Presently, practically all newly reported compounds are obtained analytically pure by chromatography. Thus, whether compounds are crystalline or liquid, of low or high molecular weight, non-polar or polar, etc., they are likely to be subjected to chromatography. The first instances of SDEvC were independently discovered by Cundy and Crooks [134] in 1983, and a year later in 1984 by Charles and Gil-Av [25], their discoveries coinciding with the beginning of wide availability of HPLC instrumentation and improved sensitivity of analyte detection. Details of these pioneering papers can be found in the following review articles [5,6,78,126]. For the purposes of the present account, we discuss a few examples dealing with routine, gravity-driven column chromatography, since it is routinely used in virtually all research laboratories for the isolation and purification of reaction products.

**Scheme 4:**
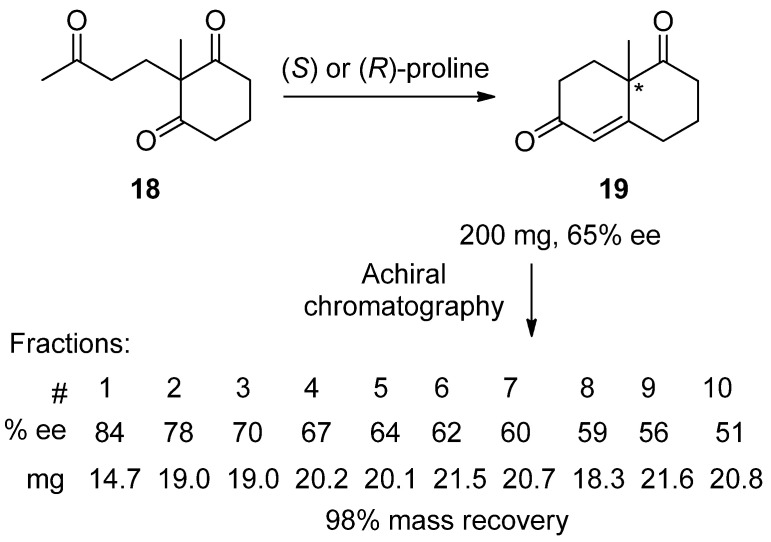
The SDEvC of product **19** by routine, gravity-driven column chromatography over silica gel using *n*-hexane–ethyl acetate (4:1) as eluent. The chiral center is marked with an asterisk in **19**.

One of the first and most remarkable examples was reported by Prof. Andre S. Dreiding [11] during the examination of the proline-catalyzed aldol cyclization of triketone **18** to bicyclic product **19** (Scheme 4). Having encountered a problem of low reproducibility of the stereochemical outcome and traced its origin to the chromatographic purification step, the authors performed a detailed investigation of the chromatographic behavior of enantioenriched diketone **19**. In a key experiment, they subjected 200 mg of **19** of 65% ee to achiral chromatography over silica gel using *n*-hexane–ethyl acetate (4:1) as an eluent, for which the ee and mass of each of the ten fractions they collected are given in Scheme 4. The authors concluded that achiral chromatography cannot be safely used for the purification of scalemates since, at least for this example, the outcome could be reported as high as 84% ee (the first fraction), as low as 51% ee (the last fraction), or anywhere in between. Pointedly, this report appeared well ahead of the “golden age” of organo-catalysis, where the chromatography of reaction mixtures to separate the products from the organo-catalyst is a typical part of the reaction work up. However, Dreiding’s warning seemingly did not have much impact on research practice, remaining practically uncited. As an exception, we are pleased to note the recent work by Prof. Eusebio Juaristi [135], which serves as an example of how high-quality organo-catalytic research should be performed and reported. For other examples of high-quality experimental work on organo-catalytic and transition metal-catalyzed reactions, other recent papers [136,137] also stand out.

**Scheme 5:**
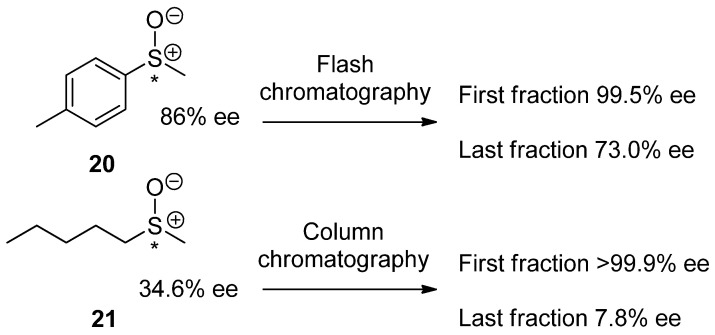
The SDEvC of compounds **20** and **21** by flash and routine, gravity-driven column chromatography, respectively. The chiral center is marked with an asterisk in **20** and **21**.

Chiral sulfoxides are in extremely high demand in academic research and the chemical industry concerned with the design and development of new synthetic reagents [138,139,140,141], drugs [142,143], and functional materials [144,145,146,147,148]. Due to the highly polar nature of the S–O bond, scalemic sulfoxides are prone to the SDEvC, rendering the sulfoxide functionality an SDE-phoric group [81]. This property is known to lead to mistakes in the evaluation of the sulfoxide enantiopurity if due care is not taken in the work up and purification steps [80,85]. The first warning of apparent difficulties caused by the SDEvC of enantioenriched sulfoxides was reported by Prof. Henri B. Kagan [38] in 1994. They demonstrated that the application of routine, achiral flash chromatography for the isolation or purification of sulfoxides can result in an erroneous description of the stereochemical outcome. For example, routine chromatography of a simple methyl *p*-tolyl sulfoxide **20** (Scheme 5) of 86% ee gave fractions of **20**, with the ee ranging from 99.5% (first) to 73% (last). The alarming SDEvC properties of chiral sulfoxides were further conveyed by Kagan et al. in a series of successive publications [119,120,149,150,151,152], but the warning failed to influence the conservative mindset of the wider research community. Thus, out of some 700+ papers reporting catalytic enantioselective synthesis of chiral sulfoxides that have been published since the seminal work, only one review [153] and four research reports [154,155,156,157] cite Kagan’s work. One might speculate on how many reports on the catalytic asymmetric synthesis of sulfoxides may contain questionable, to say the least, data.

Inspired by Kagan’s work, several investigations [26,71,80,85,102] revealed the remarkable persistence and high magnitude of the SDEvC for structurally varied chiral sulfoxides. In particular, SDE via routine, gravity-driven achiral column chromatography of methyl *n*-pentyl sulfoxide (**21**, Scheme 5) was systematically studied [85]. Parameters that were varied included the eluent, stationary phase, sample loading, temperature, and flow rate. The standout conclusion of the study was that while the SDE magnitude was strongly influenced by the nature of solvents used for elution, a noticeable value of the SDE was always observed regardless of the conditions, underscoring the strength of the intramolecular interactions leading to the persistence of the SDE. The influence of the eluent on the SDE magnitude can also be used to maximize ∆ee, allowing the development of an efficient enantiopurification method via simple column chromatography. As shown in Scheme 5, under SDE-optimized conditions, column chromatography allowed the fractionation of **21** relatively low 34.6% ee into fractions with ee’s as high as 99.9% and as low as 7.8% [85]. Considering the pronounced SDEvC properties of chiral sulfoxides, one can confidently state that most of the reports on the enantioselective synthesis of this class of compounds could be due for a re-examination in terms of the reported stereospecific outcome [71] and possible re-evaluations of the results may be in order.

**Scheme 6:**
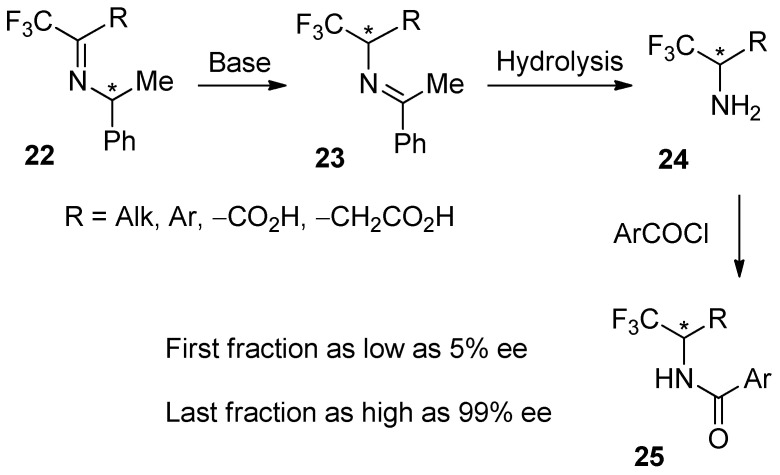
The SDEvC of trifluoromethyl-containing amides **25** by routine, gravity-driven column chromatography over silica gel using *n*-hexane–ethyl acetate (5:1) as eluent. The chiral center is marked with an asterisk in **22**–**25**.

Taking into account the ever-growing importance of fluorinated compounds in the design of modern pharmaceuticals [90,91,92,94,95,96] and agrochemicals [97,98,99,100], the trifluoromethyl-containing amino derivatives (Scheme 6) are given as the final example in this section. Thus, in the course of the development of biomimetic transamination [158], starting from either chiral imines **22** [159,160] and using a chiral based-catalyst [161,162], target amines **24** via **23** were transformed into amides **25**. However, it was found that the purification of amides **25** by routine, gravity-driven column chromatography resulted in low reproducibility of the stereochemical outcome of the reactions, prompting investigation of the underlying cause of the problem.

It was found that, similar to sulfoxides, compounds **25** also exhibit high ∆ee’s and a persistent SDE profile under a variety of chromatographic conditions. Following established methodology, the application of polar, protic solvents led to a decrease in ∆ee, while the use of aprotic, nonpolar solvents resulted in a noticeable increase in ∆ee. Under SDE-optimized conditions, routine, gravity-driven column chromatography provided enantiopure samples of amides **25**, including α- (R = –CO_2_H) [163,164] and β-amino acids (R = –CH_2_CO_2_H) [165,166], while the enantiodepleted fractions were as low as 5% ee [29,33,86]. It should be pointed out that amides **25** represent a combination of two SDE-phoric groups, –CF_3_ and –CONH–, leading to strong homo- and heterochiral intermolecular interactions—the foundation of the SDE phenomenon. Considering literature data [87,123,124,125] and our own experience in the synthesis and properties of fluoro-organic compounds, in particular, catalytic enantioselective reactions [167,168,169,170,171,172,173], we can confidently conclude that practitioners working with –CF_3_-containing compounds should be ready to encounter noticeable magnitudes of the SDEvC (i.e., ∆ee >5%).

Similar to sublimation/evaporation, chromatography is also a key basic part of typical reaction work-up and the preparation of analytical samples. Notably, SDEvC has been reported for all manner of chromatographic techniques and stationary phases, including, as exemplified in this section, routine, gravity-driven column chromatography, as commonly used in any chemistry laboratory. Considering all these facts and data, it is inconceivable to come up with an argument against a mandatory requirement for performing SDEvC tests to ensure the accuracy, quality, and consistency of reported experimental results.

## 4. Recommended SDEvS/E and SDEvC Tests

### 4.1. General Considerations

The necessity of conducting tests to gauge the SDE magnitude to ensure the veracity of reported ee’s cannot be overstated, since the SDE always occurs to some degree whenever a scalemic sample is subjected to physicochemical processes concomitant with fractionation of the sample, since even handling or storage can induce significant ee alterations to the bulk of a scalemic sample due to the SDE [10,66,79,80]. As we have documented, literature data confirm that whenever enantioenriched samples are handled, stored, or a physicochemical process applied, a test to gauge the magnitude of the SDE should be appropriately conducted, as ignorance of the SDE can lead to erroneous reporting of the ee of the sample if due care is not taken to either circumvent the SDE by measurement of the ee prior to the application of physicochemical processes, minimizing the SDE, or evaluating all obtained fractions of the sample (or avoiding fractionation altogether if possible). As outlined above, common physicochemical processes that are routinely performed in laboratories that can lead to the alteration of the ee due to the SDE, aside from obvious processes such as recrystallization and sublimation, include most, if not all, forms of achiral chromatography and even solvent evaporation under vacuum [40,44]. Indeed, the growing demand for accurate experimental data and increases in the sensitivity of analytical techniques has led to observations of the SDE in ever more processes; for example, SDE via PTLC has been reported [10,41] for the first time just recently. The important requirements for an SDE test are that it is an easy, fast, convenient, and simple procedure to perform. The main criterion for the tests is to match the conditions closely with those used in the work to evaluate the magnitude of the SDE under the applied conditions. Clearly, the major goal of proposing SDE tests via sublimation and chromatography is to ascertain the effect of the SDE and its magnitude on the compounds under study and to prevent the erroneous reporting of the stereochemical outcome. However, a secondary goal is to encourage the reporting of the SDE data collected under uniform conditions, allowing analysis, processing, and rationalization of the collected data. Additionally, we advocate the use of SDEvC for the routine preparation of enantiopure analytical samples, and thus foster a new research practice of obtaining chiral compounds as enantiopure, not simply having them as they are obtained from reactions lacking any enantioenrichment. Analogy can be seen with the chemical purity of compounds and how they evolved from as-obtained-from-the-reaction to a standard of chemical purity supported by analytical data. For the proposed SDE tests, we were careful to make the tests practical and simple to minimize resistance based on the required time, labor, and/or the cost of reagents. Having conducted the SDE test(s), this can be stated in the experimental section, along with the conclusions and the results of the SDE tests lodged in the supporting documentation.

### 4.2. SDEvS Test

The SDEvS test should be conducted for at least one representative compound of a series prepared and isolated by the same procedure. A compound bearing a substituent such as –CF_3_, *tert*-butyl, isopropyl, a long alkyl chain, or an aromatic group, if available, is best, as these groups generally impart increased volatility to organic compounds. It is suggested to use as much material as possible for the test, perhaps as much as 50 mg, to minimize weighing and other errors and, most importantly, to use analytically pure material, i.e., chemically pure and both solvent and water free, to avoid errors with the mass loss. Since the majority of the material is recovered from the test, the use of large quantities is feasible, and thus the choice of compound to be used for the test may also be determined by availability. The ee of the sample can be anywhere between 0 and 100% ee, though if available, a sample of ~50% ee is recommended to allow for similar sublimations of the racemic and enantiomeric excess portions.

Step 1. Take 50 mg of known exact mass and ee of the representative compound and leave in a flask for 12 h at 60 °C and subjected to a pressure of ~80 mbar. Afterwards, weigh the sample and in the case of scenario a, the mass is unchanged, in which case the test is completed and there are likely no complications with the SDEvS. For scenario b, the mass has decreased, in which case, measure the ee of the sample to confirm whether the mass loss has led to a decrease or increase in the ee and then proceed to Step 2.

Step 2. Conduct sublimation of the sample in a sublimation apparatus, collecting both the sublimed and residual material and measure their ee’s. In addition, other samples of the compound of differing ee should also be examined, as the ee selected may happen to be close to the euamotic point, thus samples with ee’s far from the ee of the first sample, either much higher or lower or even both, should be subjected to sublimation for a complete evaluation of the SDEvS. In this instance, it is also worth checking a sample with an ee close to the ee’s of the samples produced in the work. Other compounds from the work should also be studied and alterations to the isolation and characterization procedures should be made and the reported data adjusted accordingly. Depending on the level of interest, crystallographic data can be collected to explain the observed rates of sublimations, and obviously, any additional work could potentially be published separately as a valuable contribution to SDE research.

Though the routine measurement of mp’s seems to have fallen out of favor, it is still recommended [1], since for compounds which behave as racemic compounds by sublimation, similar mp’s for enantiopure and racemic samples of a compound will likely only result in small ∆ee’s by SDEvS [10]. While similar mp’s may provide some comfort that the SDEvS might be small in the short term, unfortunately it will not ensure that the SDEvS will be insignificant for an extended period of time under vacuum or for long-term storage. For compounds which behave as conglomerates by sublimation, the sublimate is always racemic and thus the ee of the residue can be drastically altered rather quickly with the progress of sublimation.

### 4.3. SDEvC Test

The SDEvC test should be conducted for at least one representative compound of a series prepared and isolated by the same procedure. Taking into account that intermolecular interactions and the dynamic formation of the homo- and heterochiral species are the underlying mechanism for the SDEvC, it is suggested to select a compound bearing a polar substituents such as –CF_3_, –NO_2_, –OH, –NH_2_, etc., amide, –SO, –SO_2_, acyl, or any other group that can facilitate hydrogen bonding or dipole–dipole interactions. It is suggested to use as much material as possible for the test, perhaps as much as 50 mg in the case of routine, gravity-driven column chromatography, to minimize weighing (e.g., for small quantities in collected fractions) and other errors, and to use analytically pure material, i.e., chemically pure and both solvent and water free, to avoid errors with possible mass losses. Since the majority of the material is recovered from the test, the use of large quantities is feasible, and thus, the choice of compound to be used for the test may also be determined by availability. The ee of the sample can be anywhere between 0 and 100% ee, though it is also worth testing a sample with an ee close to the ee’s of the samples produced in the work. However, if available, a sample of ~60–70% ee is preferred, as we have found that this often provides the largest ∆ee’s. The eluent, flow rate, and sample loading (relative to the amount of stationary phase) should be kept the same as in the general experimental procedure, to evaluate the effect of the SDEvC. The SDEvC is concentration dependent, so it is vitally important that lower concentrations are not used in the test compared with the general experimental procedure, and hence also why large amounts of material are recommended for the test.

Step 1. Take 50 mg of known exact mass and ee of the representative compound and subject it to chromatography using the same purification procedure as applied in the work. The elution of the compound should be fractionated with the first fraction consisting of ~5–10% of the weight, followed by three more-or-less equal fractions of ~30%, which may typically be 10 mL in volume for column chromatography, and then one final, very large fraction collected as the “tail”, which is necessarily highly diluted due to the collection of a large volume. Afterwards, the ee of all five fractions should be determined, e.g., by chiral HPLC, and in the case of scenario a, the maximum ∆ee for the fractions is less than 5% ee, in which case, one can proceed accordingly under the assumption that the influence of the SDEvC on the recorded ee’s is minimal or even insignificant and the values can be reported appropriately as ±5% ee or by whatever ∆ee value has been determined. Alternatively, the eluent can be modified by increasing the portion of the polar ingredient to suppress the intermolecular interactions, and therefore the magnitude of the SDE. The SDEvC test should then be repeated with the new eluent composition to confirm the decrease in the ∆ee. If successful, the general experimental procedure should be adjusted using the modified eluent. Of note, this adjustment of the eluent can work for SDE magnitudes far greater than 5% ee, by using protic, polar solvents such as alcohols, which have very powerful suppressive effects on hydrogen bond-based and dipole–dipole intermolecular interactions and therefore manifestation of the SDEvC. For scenario b, if ∆ee exceeds 5% ee, one should follow the experiment described in the seminal publication by Prof. Andre S. Dreiding [11]. This experiment should then be a special section in the ensuing paper, with a proper discussion on how the reported stereochemistry values were obtained, mitigating the impact of the SDEvC. Depending on the level of interest, one could follow the guidelines of paper [85] to investigate all aspects of the phenomenon, develop an SDEvC-optimized procedure, and then use the SDEvC for the preparation of enantiopure samples of the compounds under study. As stated above, a sample of ~60–70% ee often provides the largest ∆ee’s, especially when the polarity of the eluent is adjusted such that an *R*_f_ value of between 0.2–0.3 is obtained and the amount of stationary phase that is used in column chromatography is set according to a ratio of 1 mmol of compound/30 g stationary phase. Obviously, any additional work could potentially be published separately as a valuable contribution to SDE research.

## 5. Conclusions and Afterword

### 5.1. Conclusions

The importance and necessity of conducting tests to gauge the magnitude of the SDE phenomenon to ensure the veracity of reported ee’s for scalemic samples obtained from, for example, enantioselective reaction or by the isolation of natural products, has been enunciated. It has to be recognized that the SDE is always occurring to some degree whenever any scalemic sample is subjected to a physicochemical process concomitant with fractionation of the sample and the question is how to minimize—ideally, eliminate to all intents and purposes—its potentially detrimental effect on the true value of the ee. Thus, erroneous reporting of the true ee of samples can occur if due care is not taken to either preclude the effects of the SDE by measurement of the ee prior to the application of physicochemical processes, suppressing the SDE, or evaluating all obtained fractions of the sample—or even avoiding fractionation altogether if possible—and in regard to measuring the ee [4]. Thus, there is a clear necessity to conduct tests to assess the magnitude of the SDE for the processes applied to samples. However, despite a clear call for conducting SDE tests in the past [174], only a few groups [80,135,167,171,172,173,175,176,177,178,179,180,181] have taken on the recommendation, but we hope that with the updated and improved recommendations described herein, which cover chromatography and processes involving gas-phase transformations such as evaporation or sublimation, that it will become standard practice for the conduct of high-quality research involving enan-tioselective reactions, natural products, and other work involving chiral compounds.

### 5.2. Afterword

A logical consequence of a wide acceptance of the SDE and legitimization of the SDE tests, of course, would be to put into question many, if not practically all, previously reported results on the stereochemical outcome of enantioselective reactions and the stereochemical state of isolated natural products. Considering the ubiquitous nature of the SDE phenomenon and its manifestation via such standard techniques of routine work-up procedures such as evaporation, drying, and achiral chromatography, such a view is obviously valid. Accepting that some stereochemical data from the past may actually be of greater or lower ee than reported does not, however, alter or depreciate the ingenuity, innovation, and synthetic value of the developed methods. As outlined in this article, the research data on the SDE phenomenon have reached a critical mass clearly indicating the necessity for introducing SDE tests. In fact, it is impossible to find any scientifically based argument against the proposal to improve the quality of reported data, credibility of research, and public perception of science as a self-correcting entity. Bearing in mind ever-increasing budgetary constraints, limited resources, and demands on research time, we have suggested a minimum of essential tests for only one extra experiment for one representative compound to estimate the potential magnitude of the SDEvS or SDEvC. In cases of high SDE magnitude, the additional research work is not a burden, but rather, a logical extension of the original research. Thus, while the scientific aspect of the SDE tests in terms of acceptance is beyond discussion, the political thinking of the editorial boards can be an issue, as authors may naturally tend to submit and publish following a course of least resistance. However, sooner or later, it is expected that SDE tests will become obligatory and considered as standard practice in research laboratories dealing with chiral compounds.
